# Challenges of the eighth edition of the American Joint Committee on Cancer staging system for pathologists focusing on early stage lung adenocarcinoma

**DOI:** 10.1111/1759-7714.14785

**Published:** 2023-01-02

**Authors:** Yu‐Ting Wang, Il‐Chi Chang, Chih‐Yi Chen, Jiun‐Yi Hsia, Frank Cheau‐Feng Lin, Wan‐Ru Chao, Tuan‐Ying Ke, Ya‐Ting Chen, Chih‐Jung Chen, Min‐Shu Hsieh, Shiu‐Feng Huang

**Affiliations:** ^1^ Department of Anatomical Pathology Chung Shan Medical University Hospital Taichung Taiwan; ^2^ Institute of Molecular and Genomic Medicine National Health Research Institutes Miaoli Taiwan; ^3^ Department of Thoracic Surgery Chung Shan Medical University Hospital Taichung Taiwan; ^4^ Department of Pathology and Laboratory Medicine Taichung Veterans General Hospital Taichung Taiwan; ^5^ Department of Pathology National Taiwan University Hospital Taipei Taiwan

**Keywords:** adenocarcinoma in situ, AJCC staging system, lung adenocarcinoma, minimally invasive adenocarcinoma, recurrence

## Abstract

**Background:**

The eighth edition of the American Joint Committee on Cancer (AJCC) staging system for lung cancer adopts new criteria for tumor size, and for determining pTis, pT1a(mi), and pT1a. The latter is based on the size of stromal invasion. It is quite challenging for lung pathologists.

**Methods:**

All patients who had undergone surgical resection for pulmonary adenocarcinoma (ADC) at Chung Shan Medical University Hospital between January 2014 and April 2018 were reviewed, and restaged according to the eighth AJCC staging system. The clinical characteristics and survival of patients with tumor stage 0 (pTis), I or II were analyzed.

**Results:**

In total, 376 patients were analyzed. None of the pTis, pT1a(mi), or pT1a tumors recurred during the follow‐up period up to 5 years, but pT1b, pT1c, pT2a, and pT2b tumors all had a few tumor recurrences (*p* < 0.0001). In addition, 95.2%, 100%, and 77.5% of pTis, pT1a(mi), and pT1a tumors, respectively, had tumor sizes ≤1.0 cm by gross examination. All pTis, pT1a(mi), and pT1a tumors exhibited only lepidic, acinar, or papillary patterns histologically.

**Conclusions:**

This study demonstrated excellent survival for lung ADC patients with pTis, pT1a(mi), and pT1a tumors when completely excised. To reduce the inconsistencies between pathologists, staging lung ADC with tumors of ≤1 cm in size grossly as pTis, pT1a(mi), or pT1a may not be necessary when the tumors exhibit only lepidic, acinar, or papillary histological patterns. A larger cohort study with sufficient follow‐up data is necessary to support this proposal.

## Graphical abstract

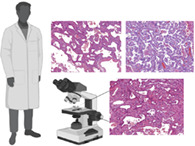

## INTRODUCTION

In 2011, the International Association for the Study of Lung Cancer (IASLC), American Thoracic Society, and European Respiratory Society proposed a multidisciplinary classification for lung adenocarcinoma (ADC).^1^ Subsequently, the IASLC initiated a lung cancer staging project to develop the eighth edition of the tumor‐node‐metastasis (TNM) classification system for lung cancer by using a retrospective and prospective database.^2^, ^3^, ^4^, ^5^, ^6^ This new classification system clarifies all aspects of clinical practice and pathology, in addition to providing diagnosis standards in clinical medical practice. Furthermore, it proposes new pathological descriptions of lung ADC. Specifically, the terms “bronchioloalveolar carcinoma” and “mixed‐subtype adenocarcinoma” should no longer be used, and instead, the two terms “adenocarcinoma in situ” (AIS) and “minimally invasive adenocarcinoma” (MIA) should be used to describe resected tumors: An AIS tumor is a small lung ADC tumor (≤3 cm) with a pure lepidic pattern and no evidence of stromal invasion. An MIA tumor is a lepidic‐predominant ADC tumor (≤3 cm) with stromal invasion but with an invasion size of ≤0.5 cm. Both terms are new concepts for small early stage ADC.^1^ When these two types of tumors are resected completely, the disease‐specific survival rate of patients is nearly 100%.^7^, ^8^ AIS and MIA were adopted in the 2015 World Health Organization (WHO) classification of lung tumors.^9^, ^10^ In 2017, the American Joint Committee on Cancer (AJCC) published the eighth edition of their Cancer Staging Manual (hereafter referred to as the eighth AJCC staging system) for non‐small cell lung cancer (NSCLC). The eighth AJCC staging system incorporates the 2015 WHO classification of lung tumors, including the AIS and MIA concepts.^11^ According to this staging system, if the stromal invasion size is >0.5 cm or if the tumor does not have lepidic patterns, then the tumor can be classified into a different T stage. This staging system was implemented in the United States in January 2018,^11^ and several other countries worldwide adopted this system in the same year. Accordingly, the system has substantially affected the staging of early NSCLCs and is critical for thoracic surgical oncologists in their daily practice.^11^

The 7th AJCC staging system and the eighth differ primarily with respect to the T component, particularly tumor size. In the eighth AJCC staging system, tumor size is based on the size of stromal invasion and not on gross examination of the whole tumor. The defined size criteria for a pT1a tumor have changed from ≤2.0 cm to >0.5 and ≤1.0 cm, and those for a pT1b tumor have changed from >2.0 and ≤3.0 cm to >1.0 and ≤2.0 cm. Moreover, pT1c is a new entity and refers to a tumor of size >2.0 and ≤3.0 cm. The size criteria for pT2a, pT2b, and pT3 tumors have also been changed. The new tumor size criteria for NSCLC are based on analyses of survival data of 15 240 patients with stage IA, IB, or IIA NSCLC (T1‐2N0 M0 R0); the analysis results demonstrated significant survival differences for every 1 cm increase in tumor size.^2^, ^3^, ^4^, ^5^, ^6^ In the seventh AJCC staging system, pTis is the only stage specified for carcinoma in situ; AIS and MIA are not included in this system. All tumor sizes in the seventh AJCC staging system are based on imaging data or gross examination results. By contrast, the new tumor size criteria in the eighth AJCC staging system, including pTis (equal to AIS), pT1a(mi) (equal to MIA), and pT1a, are based on stromal invasion sizes evaluated through microscopic examinations, which can be challenging for lung pathologists. In addition, surgeons often submit frozen sections of small tumors for pathological examination in order to determine the appropriate surgical procedure. Thus, the lung tumor is often divided into two parts, which further increases the complexity of determining the tumor size and stromal invasion size. Another challenge is the histological pattern. According to the WHO classification, tumors with any histological pattern other than lepidic cannot be classified as AIS tumors.^11^ If a tumor has a purely lepidic pattern, the tumor size is equal to the whole tumor size, which is straightforward. However, lung ADC tumors can have mixed histological subtypes, and judging whether the histological subtype is lepidic, acinar, or papillary or assessing the size of stromal invasion when the whole tumor size is ≤1 cm is complex. Thunnissen et al. reported that the reproducibility of histopathological subtypes and invasion in pulmonary ADC would decrease because of these classification changes, especially with regard to stromal invasion.^12^

With the advent of low‐dose computed tomography (CT), lung cancer screening during health examination has become a common approach in Taiwan in recent years, which could facilitate the identification of early stage lung cancer tumors with a size of ≤1 cm.^13^ Numerous small ground‐glass nodules (GGNs) in the lungs have been identified and excised for pathological examination. Thus, pathologists in each medical center could receive hundreds of small GGNs from wedge resections every year. Measuring the size of the stromal invasive component precisely for a small lung nodule is challenging. Moreover, the determination of the p pTis, pT1a(mi), and pT1a stages of lung ADC with a size ≤1 cm might be arbitrary.

Accordingly, the objective of this study was to examine the clinical significance of the 8th AJCC staging system for small lung ADC. To conduct survival analyses, we retrospectively reviewed the pathological diagnoses and outcomes of all patients who had undergone surgical resection for lung ADC between 2015 and 2018 at Chung Shan Medical University Hospital. We primarily focused on patients with stage 0, I, or II ADC without metastasis (represented by stages pTis, pT1, and pT2 and N0 M0 R0 in the eighth AJCC staging system). Clinical variables and other data, such as health examination and family history records, were also analyzed.

## METHODS

### Patients

We collected and reviewed the pathological examination reports of all patients with lung ADC who had undergone surgical resection at Chung Shan Medical University Hospital between January 2014 and April 2018. Two board‐certified pathologists (YTW and SFH) reviewed the histopathology of all patients. Another two board‐certified pathologists (CJC of Taichung Veterans General Hospital and HMS of National Taiwan University Hospital) also help to review the histopathology of the patients with the diagnosis of pTis, pT1a(mi), and pT1a. The pathological diagnoses and staging for patients operated on before 2018 were revised according to the criteria of the eighth AJCC staging system. The pTis is a lung ADC tumor (≤3 cm in size) with a pure lepidic pattern and no evidence of stromal invasion. The pT1a(mi) is a lepidic‐predominant ADC tumor (≤3 cm) with stromal invasion, but with an invasion size of ≤0.5 cm. The lung ADC of pT1a is a tumor with stromal invasion size being >0.5 cm but ≤1.0 cm. All pTis, pT1a(mi) and T1a needed to have no evidence of necrosis, lymphovascular invasion, and features of spread through air spaces (STAS).^11^, ^14^ Patients with multiple tumors were staged according to the criteria proposed by IASLC.^15^, ^16^, ^17^ Furthermore, the medical records of these patients were reviewed for further clinical analysis. The patients were divided into two groups according to smoking status: nonsmokers and ever‐smokers (comprising those who had smoked <100 cigarettes in their lifetime). This retrospective clinical data analysis was approved by the Institutional Review Board of Chung Shan Medical University Hospital (CS2‐18123).

### Statistical analysis

Chi‐square and Fisher's exact tests were performed for categorical variables. A Kaplan–Meier curve and Cox regression model were used to compare recurrence‐free survival rates among the patients at different disease stages. A linear‐by‐linear association method was used to compare the significance of several clinical variables, including sex, age, smoking history, tumor stage, family history, health examination, and tumor multiplicity. A *p*‐value of <0.05 was considered to indicate a statistically significant difference between the groups compared in the study.

## RESULTS

### Clinical characteristics

**FIGURE 1:**
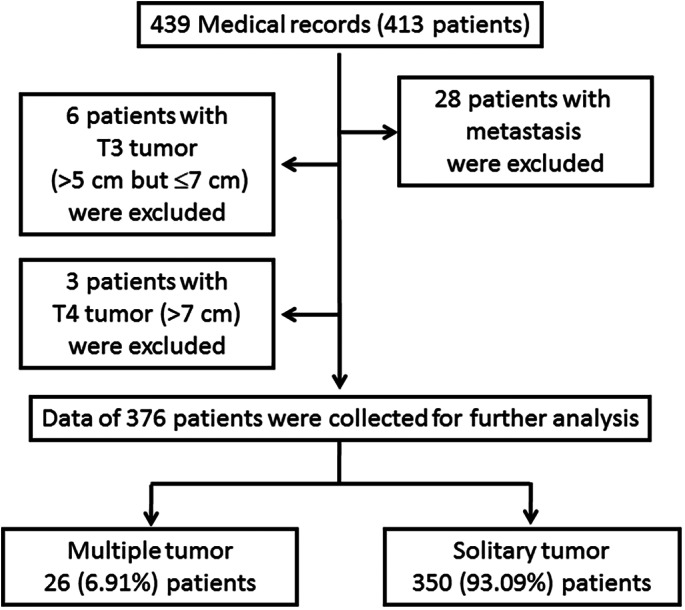
Flow chart of lung cancer patients analyzed in this study

We collected 439 pathological examination reports for 413 patients who had undergone surgical resection for lung ADC between January 2014 and April 2018. Of these patients, 28 with metastatic disease, six with pT3 tumors (tumor size >5 and ≤7 cm), and three with pT4 tumors (tumor size >7 cm) were excluded. Thus, this study included a total of 376 patients for analysis. The flow chart of the patient selection process is presented in Figure 1.

Of the 376 patients, 114 (30.32%) were men and 262 (69.68%) were women. The median (range) age was 58 (24–82) years. Of the patients, 303 (80.59%) were nonsmokers. Furthermore, 93 (25%) patients had a family history of lung cancer. Lung cancers were identified through health examinations using low‐dose CT in 196 (52.13%) patients. The clinical characteristics of the 376 patients are listed in Table 1.

**TABLE 1:** Basic clinical characteristics of the 376 lung adenocarcinoma patients

Variables	Patient number
Total patient number	376 (100%)
Age (median [min–max])	58 (24–82)
≤30	2 (0.53%)
31–40	23 (6.12%)
41–50	65 (17.29%)
51–60	127 (33.78%)
61–70	118 (31.38%)
≥71	41 (10.9%)
Sex	
Male	114 (30.32%)
Female	262 (69.68%)
Smoking	
Yes	73 (19.41%)
No	303 (80.59%)
Family history	
Yes	93 (25%)
No	279 (75%)
Unknown	4
Healthy examination with low dose CT	
Yes	196 (52.13%)
No	180 (47.87%)
Tumor stage^a^	
pTis	62 (16.49%)
pT1a(mi)	70 (18.62%)
pT1a	80 (21.28%)
pT1b	66 (17.55%)
pT1c	62 (16.49%)
pT2a	27 (7.18%)
pT2b	9 (2.39%)
Multiplicity	
Yes (multiple)	26 (6.91%)
No (solitary)	350 (93.09%)
Recurrence	
Yes	11 (2.93%)
No	365 (97.07%)

Abbreviation: CT, computed tomography.

^a^ According to the eighth edition of the AJCC staging system.

We grouped the 376 patients into seven T stages according to the eighth AJCC staging system: pTis (62 patients), pT1a(mi) (70 patients), pT1a (80 patients), pT1b (66 patients), pT1c (62 patients), pT2a (27 patients), and pT2b (9 patients). The T stages data were established according to the consensus of the four pathologists (YTW, SFH, CJC, and HMS).

The median (range) follow‐up period for the patients was 1618.7 days (9–2951 days). Only 12 patients' follow‐up periods were shorter than 50 days (due to loss to follow‐up). With regard to the mean and median (range) follow‐up days for the patients with different stage, this is shown as follows (mean; median [range] in days): pTis (1633.2; 1590.5 [11–2445]), pT1a(mi) (1777.4; 1866 [20–2885]), pT1a (1643.3; 1624 [13–2610]), pT1b (1536.7; 1588.5 [9–2518]), pT1c (1640.1; 1690 [18–2951]), pT2a (1411.7; 1568 [41–2558]), and pT2b (1138.6; 760 [41–2739]).

The whole tumor sizes by gross examination were also analyzed according to the pathological reports. Among the 62 patients in the pTis group, only 3 (4.8%) had a whole tumor size of >1 cm. By contrast, all of the 69 patients in the pT1a(mi) group had a whole tumor size of ≤1 cm. Furthermore, of the 80 patients in the pT1a group, only 18 (22.5%) had a tumor size of >1 cm. Regarding surgical procedures performed, wedge resection was performed in 60 (96.77%), 66 (94.20%), and 62 (77.5%) patients in the pTis, pT1a(mi), and pT1a stages, respectively. The remaining patients received lobectomy. Lymphadenectomy was performed in all patients.

**FIGURE 2:**
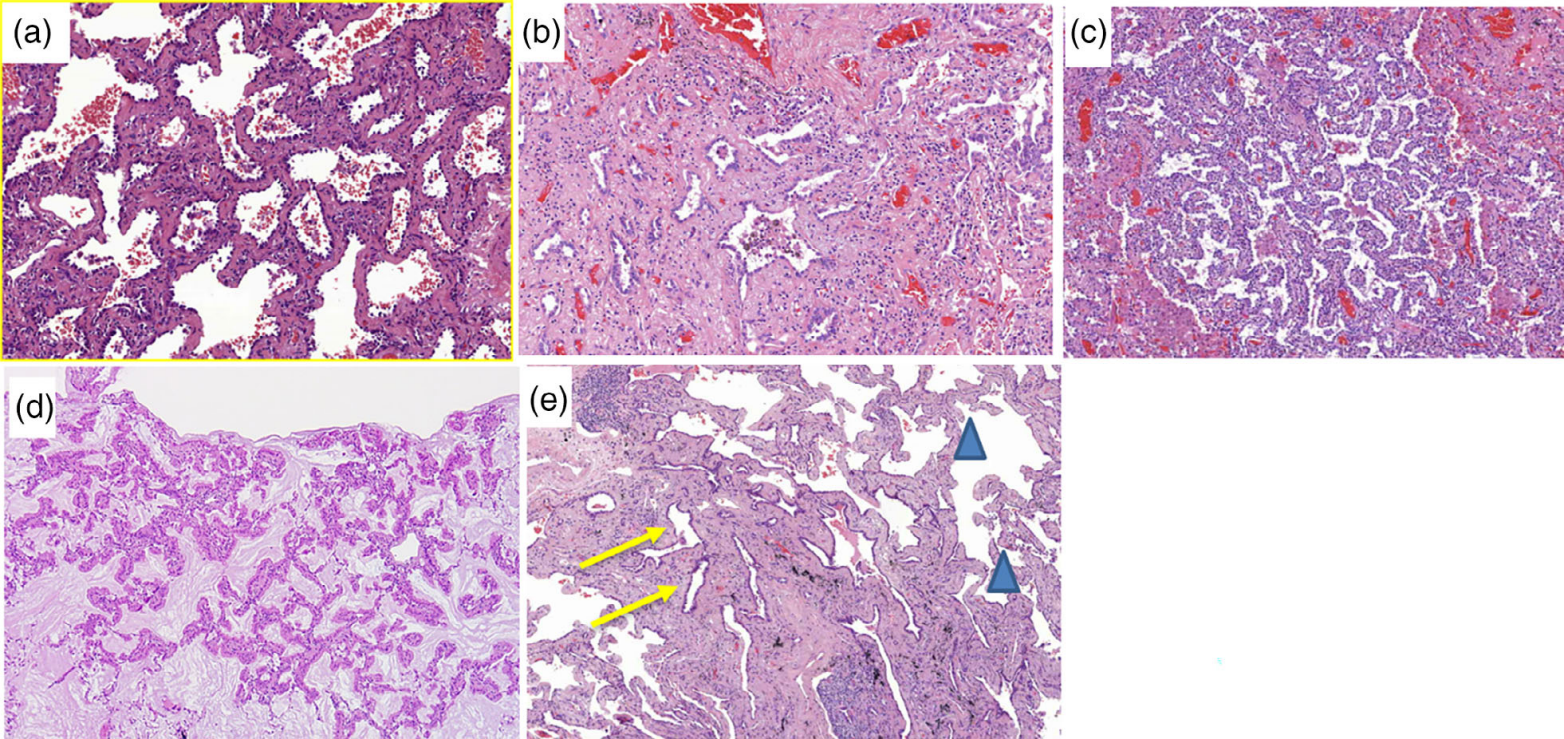
Histopathology patterns of early stage lung adenocarcinoma (all hematoxylin & eosin [H&E] stain). (a) Lepidic pattern (200×). A tumor of 7 mm in size grossly. It was diagnosed as pTis as it was composed of a lepidic pattern. (b) Acinar pattern (100×). A tumor of 7 mm in size grossly with a focal acinar pattern. It was diagnosed as pT1a(mi) as it had a mixed lepidic and acinar pattern, the latter component was <5 mm in size. (c) Papillary pattern (100×). A tumor mainly composed of a papillary pattern was 5 mm in size and was diagnosed as pT1a(mi) as it had a papillary pattern and was ≤5 mm. (d) An invasive mucinous adenocarcinoma of 6 mm in size arranged in both lepidic and papillary patterns (40×). It was diagnosed as stage pT1a. (e) A tumor of 6 mm in size with mixed histological patterns (100×). The peripheral region had a lepidic pattern (arrowheads). The central portion had fibrotic stroma with some dilated glands (arrows). Acinar or lepidic pattern with dilated lumen and thick fibrous septa were both possible and it was diagnosed as pTis

For the patients in the pT1a(mi) and pT1a stages, other than the typical lepidic patterns (Figure 2a), the histopathological features that were representative of stromal invasion were predominantly acinar patterns (Figure 2b); two of them were papillary patterns (Figure 2c), and one was invasive mucinous ADC with both lepidic and papillary patterns (Figure 2d). No solid or micropapillary patterns were observed. Tumors with mixed histology patterns were quite common. However, the judgment between acinar and lepidic pattern with dilated lumen and thick fibrous septa could be difficult (Figure 2e).

### Survival analysis

**FIGURE 3:**
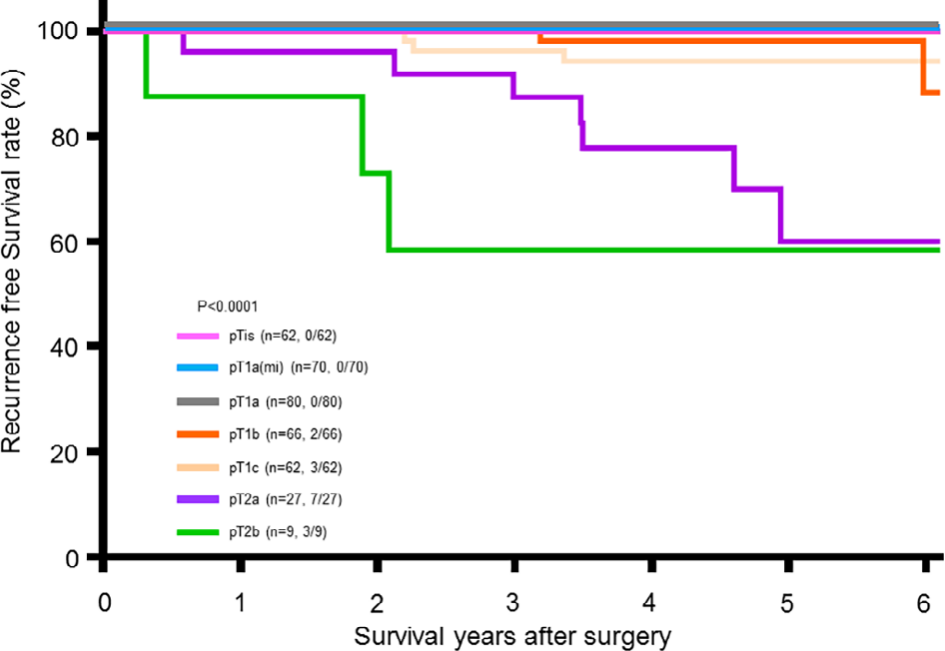
Six year recurrence‐free survival rate in 376 lung cancer patients. At each pathology T stage, total patients and fraction of recurrent patients over total patients are shown in parentheses. Six year recurrence free survival rates of stage pTis to pT1a were kept at 100%

**FIGURE 4:**
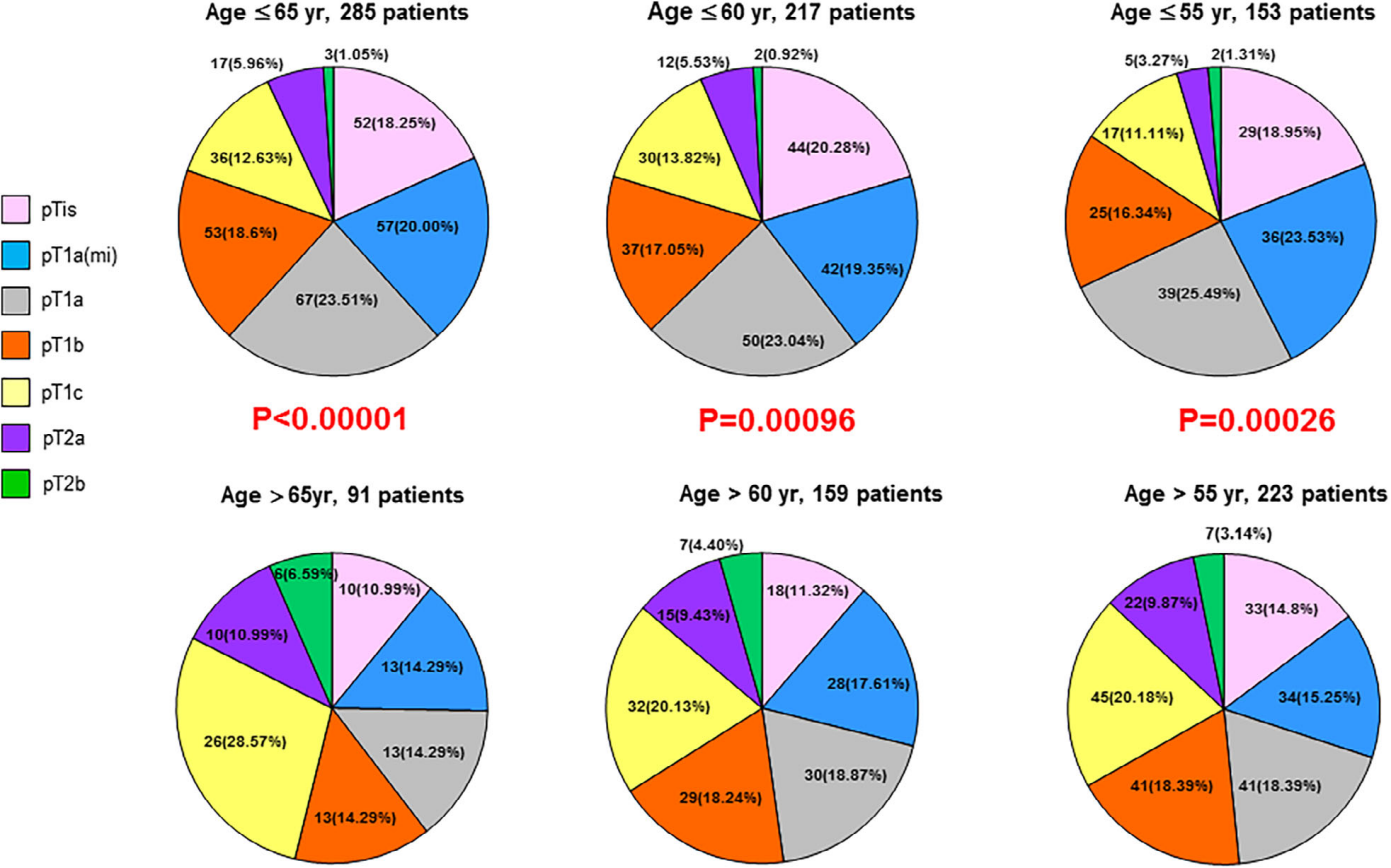
Stage distribution of 376 patients with three different cutoff ages. In each cutoff age group, older patients were associated with more advanced pathology T stages

**FIGURE 5:**
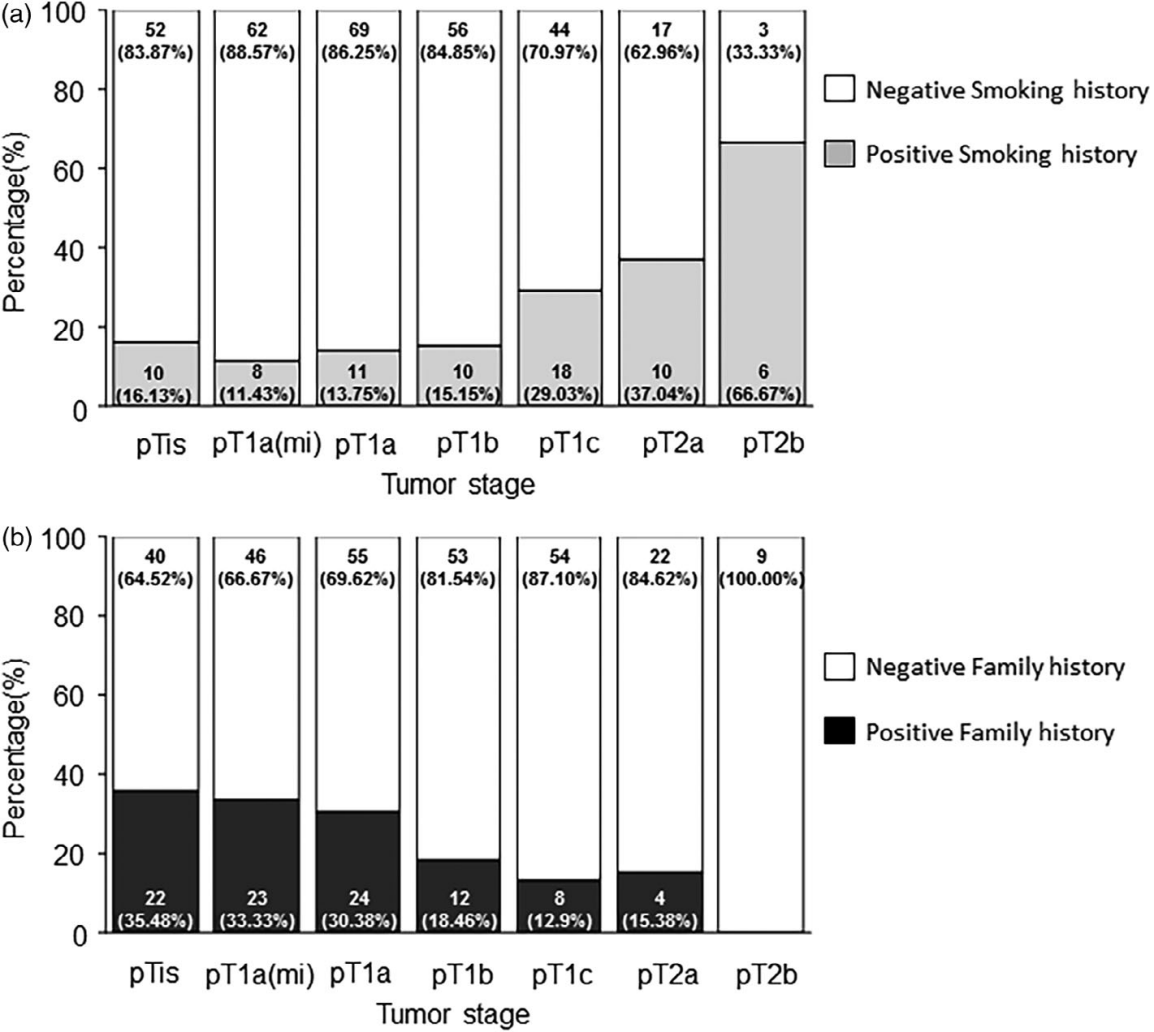
Lung cancer pathology T stage correlated with (a) smoking history (*n* = 376, *p* < 0.0000) and (b) family history (*n* = 372, *p* < 0.0000)

Only 15 (3.98%) patients had tumor recurrence by their final follow‐up date. None of the 376 patients died. Moreover, none of the patients in the pTis, pT1a(mi), and pT1a stages had tumor recurrence during the follow‐up period, whereas all the patients in the pT1b, pT1c, pT2a, and pT2b groups had some tumor recurrence during the follow‐up period up to 5 years. These results indicate that tumor stage is significantly associated with recurrence‐free survival (*p* < 0.0001; Figure 3). The annual recurrence rates for each T stage are listed in Table 2. As tumor stage is significantly associated with survival, we analyzed the association of tumor stage distribution with the various clinical variables (including age, sex, smoking status, tumor multiplicity, and family history). We observed that age was an essential factor. Regardless of whether the cutoff age was 55 (*p* = 0.00026), 60 (*p* = 0.00096), or 65 (*p* < 0.00001) years, older age was associated with more advanced tumor stages (Figure 4). Sex (*p* = 0.1140) and tumor multiplicity (*p* = 0.6361) exhibited no significant association with tumor stage distribution. Smoking status was also associated with advanced tumor stages (*p* < 0.0000; Figure 5a). Additionally, a positive family history was associated with earlier tumor stages (*p* < 0.0000; Figure 5b).

**TABLE 2:** The recurrence rate of the 376 lung adenocarcinoma patients divided by tumor stage

	Recurrence/total patient (%)						
Stage	pTis	pT1a(mi)	pT1a	pT1b	pT1c	pT2a	pT2b
Patient number	62	70	80	66	62	27	9
Follow‐up year							
<1	0/62 (0%)	0/70 (0%)	0/80 (0%)	0/66 (0%)	0/62 (0%)	1/27 (3.7%)	1/9 (11.11%)
≥1 and <2	0/60 (0%)	0/68 (0%)	0/76 (0%)	0/59 (0%)	0/58 (0%)	0/23 (0%)	1/6 (16.67%)
≥2 and <3	0/59 (0%)	0/66 (0%)	0/74 (0%)	0/58 (0%)	2/53 (3.77%)	2/23 (8.7%)	1/5 (20%)
≥3 and <4	0/57 (0%)	0/64 (0%)	0/71 (0%)	1/55 (1.82%)	1/51 (1.96%)	2/20 (10%)	0/4 (0%)
≥4 and <5	0/46 (0%)	0/57 (0%)	0/64 (0%)	0/51 (0%)	0/44 (0%)	2/15 (13.33%)	0/4 (0%)
≥5	0/20 (0%)	0/38 (0%)	0/26 (0%)	1/18 (5.56%)	0/24 (0%)	0/6 (0%)	0/3 (0%)
Overall recurrence rate	0/62 (0%)	0/70 (0%)	0/80 (0%)	2/66 (3.03%)	3/62 (4.84%)	7/27 (25.93%)	3/9 (33.33%)

Regarding the surgical procedures, the recurrence‐free survival up to 5 years for the pTis, pT1a(mi), and pT1a were all the same (all were 100%), irrespective of whether the patients received wedge resection or lobectomy.

### Impact of health examination

In this study cohort, the lung cancer diagnoses of 196 (52.13%) patients were made through low‐dose CT during health examinations. We further analyzed the association of clinical characteristics with health examination (Table 3). It showed that health examination was significantly associated with a younger age (*p* = 0.0009), early tumor stage (*p* = 0.0034), and low recurrence rate (*p* = 0.0082), but not with sex (*p* = 0.3601) or smoking status (*p* = 0.8852).

**TABLE 3:** Comparison between the 376 lung cancer adenocarcinoma patients with healthy examinations or not

Variables	Healthy examination	No healthy examination	*p*‐value^a^
Total patient no.	196 (52.13%)	180 (47.87%)	
Sex			0.3601
Male	64 (32.65%)	50 (27.78%)	
Female	132 (67.35%)	130 (72.22%)	
Age (median [min‐max])	56 (24–78)	60 (31–82)	0.0009
Smoking			0.8852
Yes	37 (18.88%)	36 (20%)	
No	159 (81.12%)	144 (80%)	
Family history^b^			0.0632
Yes	57 (29.08%)	36 (20%)	
No	138 (70.41%)	141 (78.33%)	
Multiplicity			0.8557
Yes	14 (7.14%)	12 (6.67%)	
No	182 (92.86%)	168 (93.33%)	
Stage			0.0034
pTis	38 (19.39%)	24 (13.33%)	
pT1a(mi)	37 (18.88%)	33 (18.33%)	
pT1a	48 (24.49%)	32 (17.78%)	
pT1b	35 (17.86%)	31 (17.22%)	
pT1c	28 (14.29%)	34 (18.89%)	
pT2a	8 (4.08%)	19 (10.56%)	
pT2b	2 (1.02%)	7 (3.89%)	
Recurrence			0.0082
Yes	1 (0.51%)	9 (5%)	
No	195 (99.49%)	171 (95%)	

^a^ The *p*‐value for variables other than age and stage were determined by χ^2^‐test or Fisher's exact test (for patient numbers less than 5). The *p*‐value for age and stage were determined by Mann–Whitney test.

^b^ Four patients (1 received healthy examination and 3 were not) without available family history data were not included.

### Clinical characteristics of patients with multiple lung tumors

Of the 376 patients in the study, 26 had multiple lung tumors. The clinical characteristics of these 26 patients were compared with those of the 350 patients with solitary lung tumors (Table 4). Up to 73% of the 26 patients with multiple ADC tumors had earlier tumor stages (pTis, pT1a[mi], and pT1a), whereas only 55.14% (193 of 350) of those with solitary ADC tumors had such stages; nevertheless, the difference was not significant because of the small case numbers. The female sex and nonsmoking status were also significantly associated with multiple ADC tumors; this is because 25 patients with multiple ADC tumors were women and only one was a man. In addition, of the 26 patients with multiple ADC tumors, eight had a family history of lung cancer. Of these eight patients, six had mothers with lung cancer; this association was significantly different from that observed for patients with solitary ADC tumors and a family history of lung cancer (*p* = 0.0077).

**TABLE 4:** Comparison between the 376 lung adenocarcinoma patients with solitary or multiple tumors

Variables	Multiple ADC	Solitary ADC	*p*‐value
Total patient number	26 (6.91%)	350 (93.09%)	
Age (median [min‐max])	59.5 (39–76)	58 (24–82)	0.6122
Sex			0.0014
Male	1 (3.85%)	113 (32.29%)	
Female	25 (96.15%)	237 (67.71%)	
Smoking			0.0385
Yes	1 (3.85%)	72 (20.57%)	
No	25 (96.15%)	278 (79.43%)	
Family history with lung cancer^a^			0.6386
Positive	8 (30.77%)	85 (24.57%)	
Negative	18 (69.23%)	261 (75.43%)	
Mother with lung cancer^b^			0.0077
Yes	6 (75%)	21 (25.3%)	
No	2 (25%)	62 (74.7%)	
Healthy examination			0.8557
Yes	14 (53.85%)	182 (52%)	
No	12 (46.15%)	168 (48%)	
Stage			0.6361
pTis	3 (11.54%)	59 (16.86%)	
pT1a(mi)	5 (19.23%)	65 (18.57%)	
pT1a	11 (42.31%)	69 (19.71%)	
pT1b	2 (7.69%)	64 (18.29%)	
pT1c	2 (7.69%)	60 (17.14%)	
pT2a	3 (11.54%)	24 (6.86%)	
pT2b	0 (0%)	9 (2.57%)	
Recurrence			0.5160
Yes	1 (3.85%)	9 (2.57%)	
No	25 (96.15%)	341 (97.43%)	

Abbreviation: ADC, adenocarcinoma.

^a^ In patients with solitary ADC, four patients without available family history data were not included.

^b^ In patients with solitary ADC, two patients with positive family history but whose relatives were unknown were not included.

## DISCUSSION

This study included 376 patients for analysis, and many of them had a whole tumor size of ≤1.0 cm. The reason for this is that up to 196 patients (52.13%) were diagnosed through health examinations that included low‐dose CT. None of the patients in the pTis, pT1a(mi), and pT1a stages had tumor recurrence during the follow‐up periods. Because of the favorable prognosis and the challenge of achieving an accurate evaluation of a stromal invasion size of ≤5 mm through histological examination, we suggest that staging a whole lung ADC tumor measuring ≤1 cm as pTis, pT1a(mi), and pT1a may not be essential if the tumor exhibits only lepidic, acinar, or papillary histological patterns.

The significance of staging a lung ADC tumor as pTis, pT1a(mi), and pT1a has been previously challenged. Behera et al. pooled 19 studies on AIS and MIA conducted between 2011 and 2015 and analyzed survival rates.^7^, ^8^, ^18^, ^19^, ^20^, ^21^, ^22^, ^23^, ^24^, ^25^, ^26^, ^27^, ^28^, ^29^, ^30^, ^31^, ^32^, ^33^, ^34^, ^35^ They revealed that the 5‐year disease‐free survival rates for AIS and MIA were both 100% and that the 5‐year overall survival rates for AIS and MIA were 100 and 98.5%, respectively;^18^ however, the difference was not statistically significant. Thus, they recommended further research to address questions relating to the value of the new classification system.^18^

The results of studies on the prognostic value of AIS and MIA have varied.^36^, ^37^ This is probably due to discrepancies in histology interpretations between the studies because the diagnoses of AIS, MIA, and T1a in the eighth edition of the AJCC staging system are based on pathological examination results. Thunnissen et al. performed an international interobserver study on the reproducibility of histopathological subtypes and invasion in pulmonary ADC.^12^ The authors revealed that for five mixed histological subtypes, the average kappa scores (±standard deviation) for typical patterns and difficult cases were 0.77 ± 0.07 and 0.38 ± 0.14, respectively. Regarding invasion, the kappa values for typical and difficult cases were 0.55 ± 0.06 and 0.08 ± 0.02, respectively. Therefore, their study demonstrated that in lung ADC, the interobserver reproducibility of histopathological subtypes was very low, especially for difficult cases.^12^ Furthermore, determining whether the histological pattern is lepidic, acinar, or papillary in such small ADC tumors is also challenging.^38^, ^39^ One example is the differences between the AIS by WHO lung adenocarcinoma classification^9^, ^10^ and Noguchi classification published in 1995.^40^ In the Noguchi classification for small early lung ADC (<2 cm), the 5‐year survival rates for patients with type A and type B patterns were 100%. Therefore, Noguchi et al. concluded that types A and B lesion were thought to be in situ peripheral adenocarcinoma.^40^ The histology pattern of Noguchi type A was similar to the lepidic pattern, but for Noguchi type B, the fibrous foci due to alveolar collapse could be similar to the acinar pattern, and it might be considered as having stromal invasion by some pathologists.^12^ Thus, evaluations of the size or presence of stromal invasion could be subjective, which could explain the differences in the numbers of patients classified as having stage pTis, pT1a(mi), and pT1a disease between the various studies. By contrast, the gross tumor size measurement, used in the seventh edition of the AJCC staging system, is more objective and reliable.

In the present study, the survival rates of the patients in the pTis, pT1a(mi), and pT1a stages were favorable, with the majority of the patients having a tumor size of ≤1 cm despite having only undergone a wedge resection. Accordingly, we support the conclusion of Behera et al. who challenged the necessity of dividing lung ADC into stages pTis, pT1a(mi), and pT1a.^17^ In Taiwan, the National Health Insurance program only provides reimbursement for patients with stage pT1a(mi) and pT1a lung cancer, not for patients with pTis; however, establishing a clear cutoff between 5‐ or 6‐mm stromal invasion through microscopic examination is a challenge for attending pathologists and also stressful for them.

Regarding the surgical procedures, this study demonstrated that the survival for the pTis, pT1a(mi), and pT1a were all the same (100% recurrence‐free survival up to 5 years), irrespective of whether they received wedge resection or lobectomy, which were similar to previous reports for early staged NSCLC.^41^, ^42^ The main reason for choosing wedge resection instead of segmentectomy or lobectomy was to reduce postoperative complications, especially for patients who were unsuitable for lobectomy.^41^, ^42^

In this study, eight patients had multiple lung ADC tumors, and six had mothers with a history of lung cancer; this association was significantly different from that observed for patients with solitary tumors and a family history of lung cancer. This association has not previously been reported. We identified only one study revealing that children have an increased risk of lung cancer if their mothers had lung cancer, but this association was not observed for those whose fathers had lung cancer.^43^

We also observed a clear influence of health examination on patient characteristics. Specifically, we observed that health examination was significantly associated with a younger age (*p* = 0.0009), early tumor stage (*p* = 0.0034), and low recurrence (*p* = 0.0082). Older age was associated with more advanced tumor stages regardless of whether the cutoff age was 55 (*p* = 0.00026), 60 (*p* = 0.00096), or 65 (*p* < 0.00001) years. This could be attributed to the lower rate of health examinations and to other factors. A family history of lung cancer was associated with early tumor stage (*p* < 0.0000) but not with health examination (*p* = 0.0632). This could be attributed to these patients' awareness of the importance of seeking medical care.

Smoking was significantly associated with advanced tumor stages, although the health examination rate did not differ significantly between ever‐smokers and nonsmokers. This association is similar to the results of a large cohort study that was conducted in 2010 by Kawaguchi et al. on 26 957 patients with NSCLC.^44^ The clinical variables associated with improved survival in that study included good performance status, the female sex, never smoking, early disease stage, and squamous cell carcinoma histology. Furthermore, they demonstrated that most smokers were at advanced tumor stages.^44^

In conclusion, this study demonstrated excellent survival for lung ADC patients with pTis, pT1a(mi), and pT1a tumors when completely excised. Staging lung ADC with tumors of ≤1 cm in size grossly as pTis, pT1a(mi), or pT1a may not be necessary when the tumors exhibit only lepidic, acinar, or papillary histological patterns. It is probably better to classify all of the above tumors as T1a. This proposal would have no change in the survival outcome since the recurrent free survivals up to 5 years were 100% as shown in this study, but it could reduce a lot of personal bias for Tis, T1a(mi), and T1a between different pathologists. However, the case number in this study is too small to suggest a new staging classification. A larger cohort study with sufficient follow up data is necessary to support this proposal.

## AUTHOR CONTRIBUTIONS

Yu‐Ting Wang and Il‐Chi Chang, Shiu‐Feng Huang: conducted the data analyses and wrote the manuscript. Ya‐Ting Chen: prepared the figures and tables for the manuscript. Wan‐Ru Chao, Tuan‐Ying Ke, Frank Cheau‐Feng Lin, Jiun‐Yi Hsia, and Chih‐Yi Chen: provided the research data. All authors read and approved the final manuscript.

## CONFLICT OF INTEREST

All authors have no conflict of interest.

## ETHICAL APPROVAL

This retrospective clinical data analysis was approved by the Institutional Review Board of Chung Shan Medical University Hospital (CS2‐18123).
